# Differing deregulation of HER2 in primary gastric cancer and synchronous related metastatic lymph nodes

**DOI:** 10.1186/1746-1596-8-191

**Published:** 2013-11-21

**Authors:** Mitsugu Kochi, Masashi Fujii, Shinobu Masuda, Noriaki Kanamori, Yoshiaki Mihara, Tomoya Funada, Hidenori Tamegai, Megumu Watanabe, Hiroshi Suda, Tadatoshi Takayama

**Affiliations:** 1 Department of Digestive Surgery, Nihon University School of Medicine, 30-1OHyaguchi Kamimachi, Itabashi-ku, Tokyo 173-8610, Japan; 2 Department of Pathology, Nihon University School of Medicine, Tokyo, Japan

**Keywords:** Gastric cancer, HER2, Lymph node metastasis, Primary tumor

## Abstract

**Background:**

The aim of this study was to investigate how differences in expression of HER2 between primary gastric cancers (PGCs) and their corresponding metastatic lymph nodes (LMNs) might affect its potential as a prognostic indicator in treatments including anti-HER2 agents.

**Methods:**

The analysis was conducted in 102 patients who underwent surgical resection for primary gastric cancers (PGCs; adenocarcinoma, intestinal type) with synchronous LNMs. HER2 gene status and protein expression were investigated by immunohistochemistry (IHC) in all patients; fluorescence *in situ* hybridization (FISH) was performed in 22 patients. The correlation between HER2 gene status in PGCs and their LNMs was evaluated.

**Results:**

Positive HER2 expression as detected by IHC + FISH was observed in 27/102 PGC samples (26.5%) and 29/102 LNM samples (28.4%). HER2 amplification status in 102 paired PGC and LNM samples as evaluated by FISH + IHC was concordant in 92 patients (90.2%), 69 (67.6%) were unamplified and 23/102 (22.5%) were amplified at both sites, and discordant in 10 patients (9.8%), 4 (3.9%) were positive for PGC and negative for LNM, while 6 (5.9%) were positive for LNM and negative for PGC. The results of FISH + IHC showed very strong concordance in HER2 status between the PGC and LNM groups (*k* = 0.754).

**Conclusion:**

The high concordance between HER2 results for PGCs and their LNMs indicates that assessment of HER2 status in the primary cancer alone is a reliable basis for deciding treatment with anti-HER2 agents in patients with LNMs from gastric adenocarcinoma.

**Virtual slides:**

The virtual slide(s) for this article can be found here: http://www.diagnosticpathology.diagnomx.eu/vs/9365749431029643.

## Background

A large number of studies have shown that HER2-positivity, which is associated with poor prognosis, is detected in 7% to 34% of patients with gastric cancer [1-9]. Moreover, recent advances in technology have made detection of HER2 much easier, thus increasing its potential as a prognostic marker [10,11].

Currently, clinical trials of trastuzumab and lapatinib are being conducted based on the findings of earlier preclinical *in vitro* and *in vivo* studies demonstrating that they were effective in different gastric cancer models.

Trastuzumab, a monoclonal antibody against human epidermal growth factor receptor 2 (HER2; also known as ERBB2), in combination with chemotherapy is considered a new standard option in patients with HER2-positive advanced gastric or gastro-esophageal junction cancer [12]. The efficacy of trastuzumab in metastatic gastric cancer may be influenced by downstream deregulation of HER2 signaling proteins detected in the primary tumor.

These earlier data were obtained by analyzing the clinical response in gastric cancer patients with regard to molecular features detected in the primary gastric cancers (PGCs). However, metastases may have molecular patterns which differ from those of the primary tumor, and this might affect how accurately we can predict the efficacy of HER2-targeted therapy. Differences in HER2 expression between primary tumors and their lymph node metastases (LMNs) could not explain the high proportion of non-responders to trastuzumab therapy in breast cancer [13-15]. However, the level of expression of a particular molecular marker or gene status may differ between a PGC and its corresponding metastatic lesions, and this may affect the clinical significance of predictive tests.

The purpose of the present study was to determine how differences in expression of HER2 between PGCs and their corresponding LMNs might affect its potential as a prognostic indicator in treatments including anti-HER2 agents.

## Material and methods

### Patient population

Between January 1990 and December 2010, 102 patients underwent gastrectomy for PGCs histopathologically classified as adenocarcinoma, intestinal type together with dissection of synchronous LNMs at the Department of Digestive Surgery, Nihon University School of Medicine Itabashi Hospital. The analysis was conducted in PGCs with synchronous LNMs. Tissue specimens were obtained from both the PGCs and their associated LNMs. All the specimens were fixed in 4% neutral buffered formalin and embedded in paraffin before being sent to a local pathology facility for evaluation. This study was approved by the Research review board, Nihon University School of Medicine, Itabashi Hospital (No RK-100910).

### Molecular analyses

All the specimens obtained were reviewed for quality and tumor content. A single representative tumor specimen from each patient containing at least 70% neoplastic cells was selected for immunohistochemical, cytogenetic, and molecular analyses. The tumor blocks were cut into 3-μm thick sections for analysis by both fluorescence *in situ* hybridization (FISH) and immunohistochemistry (ICH).

### Assessment of HER2 status by IHC

The tumors were centrally tested by ICH for HER2 status (HercepTest; Dako, Kyoto, Japan). HER2 immunoreactivity was evaluated by an experienced pathologist according to the scoring system of Hofmann et al. [16]. Samples exhibiting strong, complete, basolateral or lateral membranous reactivity (3+) in ≥10% of the cells were scored as positive. Samples with no reactivity or membranous reactivity in <10% of the cells, or with only faint or barely perceptible membranous reactivity (1+) in ≥10% of the tumor cells (cells showing reactivity in only one area of their membrane) were considered negative. Samples showing weak-to-moderate, complete, basolateral or lateral membranous reactivity (+2) in ≥10% of the tumor cells were scored as equivocal. Patients were eligible if their tumor samples were scored as 2+ on IHC, or if the samples were FISH-positive.

### Assessment of HER2 status by FISH

HER2 amplification was assessed in histological samples of both PGCs and LNMs by using a Spectrum Green fluorophore-labeled α-satellite DNA probe for chromosome 17 (CEP17) and a Spectrum Orange fluorophore-labeled DNA probe for the HER2 gene locus (VysisPathVysionHER2 DNA Probe Kit; Vysis-Abbott, Japan). Slides were hybridized using the Hybrite denaturation/hybridization system for FISH (Vysis). Chromosome 17 polysomy was defined as ≥3 CEP17 signals on average per cell. Amplification was defined as an HER2/CEP17 ratio of ≥2, or when an HER2 signal cluster was observed.

### Statistical analysis

Concordance between HER2 status in the primary tumor and its related metastatic sites was evaluated using Cohen’s κ-test, which is appropriate for assessing concordance between two categorical measurements in the same individual. A *κ*-value of between 0.61 and 0.8 was assumed to indicate a very strong agreement. A *P* value of less than 0.05 was considered to indicate statistical significance. The SAS software package for Windows, version 8.02 (SAS Institute Inc., Cary, NC, USA), and Microsoft Excel 2003 (Microsoft Co., Ltd., Japan) were used for the statistical analysis and data calculation.

## Results

Patient characteristics are summarized in Table 1. The 102 patients included 82 men (80.4%) and 20 women (19.6%). The median age at the time of diagnosis was 68 years (ranging from 29 to 86 years). Upper gastric cancer was present in 27 patients and middle and lower gastric cancer in 75. All patients were classified as having adenocarcinoma, intestinal type tumors. The median number of metastatic lymph nodes was 2 greater in the male group than that in the female group (4[1-100] vs. 2[1-10]respectively; *P* = 0.013). No statistically significant difference was observed in any clinical feature between males and females apart from in number of metastatic sites. The HER2 gene copy number was evaluated by IHC in 102 consecutive primary gastric adenocarcinoma specimens and their corresponding metastatic lesions and by FISH in 22. All 102 PGC and LNM specimens were adequate for IHC evaluation of HER2 status. Of the 23 PGC specimens and 23 LNM specimens selected for FISH evaluation, specimens from 1 patient were not suitable due to poor tissue fixation.

**Table 1:** Patient demographics and tumor characteristics

	**Total (%)**	**Male (%)**	**Female (%)**	
**Clinical features**	**N = 102**	**N = 82**	**N = 20**	***P *** **value**
**Sex**				
Male	82 (80.4)			
Female	20 (19.6)			
**Age (Year)**				0.348
Median [Range]	68 [29-86]	68 [29-84]	72[29-86]	
29-49	18 (17.6)	16 (19.5)	2 (10.0)	
50-79	73 (71.6)	59 (57.8)	14 (70.0)	
79-86	11 (10.8)	7 (8.5)	4 (20.0)	
**Location**				0.868
Upper	27 (26.5)	22 (26.8)	5 (33.3)	
Middle + Lower	75 (73.5)	60 (73.2)	15 (66.6)	
**Operation method**				0.454
Partial	64 (62.7)	50 (61.0)	14 (70.0)	
Total	38 (37.3)	32 (39.0)	6 (30.0)	
**Curability**				0.147
No residual tumors	87 (85.3)	72 (87.8)	15 (75.0)	
Definite residual tumor	15 (14.7)	10 (12,2)	5 (25.0)	
**Clinical stage (TNM)**				0.563
Stage I-III	82 (80.4)	65 (79.3)	17 (85.0)	
Stage IV	20 (19.6)	17 (20.7)	3 (15.0)	
**Histological stage (TNM)**				0.936
Stage II-III	86 (84.3)	69 (84.2)	17 (85.0)	
Stage IV	16 (15.7)	13 (15.8)	3 (15.0)	
**Histological type**				
Intestinal	141 (100)	82 (100)	20 (100)	
Diffuse	0 (0)	0 (0)	0 (0)	
**No of LN metastases**				0.013
Median [Range]	4 [1-100]	4 [1-100]	2 [1-10]	

### Assessment of HER2 status by IHC

HER2 expression as detected by IHC was positive or equivocal in 32 out of 102 PGC specimens (31.4%) and 34 out of 102 LNM specimens (33.3%). Overall, the general pattern of HER2 protein expression in each PGC was the same as in its corresponding LNMs (Table 2). HER2 amplification status was evaluable in 102 paired PGC and LNM specimens: 26 (25.5%) were amplified and 62 (60.8%) were unamplified at both sites. There were 6 discordant cases (5.9%), where HER2 status was positive or equivocal in PGC but negative in the LNM, and 8 discordant cases (7.8%), where HER2 status was positive or equivocal in the LNM but negative in the PGC. The results of IHC showed strong concordance in HER2 status between the PGC and LNM groups (*k* = 0.629).

**Table 2:** Comparison of HER2 status as assessed by IHC in 102 primary gastric carcinomas and their corresponding lymph node metastases

		**LN metastatic site**							
		**Negative**	**%**	**Equivocal (2+)**	**%**	**Positive**	**%**	**Total**	**%**
		**(0, 1+)**				**(3+)**			
**Primary tumor**									
Negative	(0, 1+)	62	60.8	6	5.8	2	2.0	70	68.6
Equivocal	(2+)	2	2.0	2	2.0	3	2.9	7	9.6
Positive	(3+)	4	3.9	4	3.9	17	16.7	25	24.5
Total		68	66.6	12	11.8	22	21.6	102	100

*k* = 0.629.

### Assessment of HER2 status by FISH + IHC

**Figure 1:**
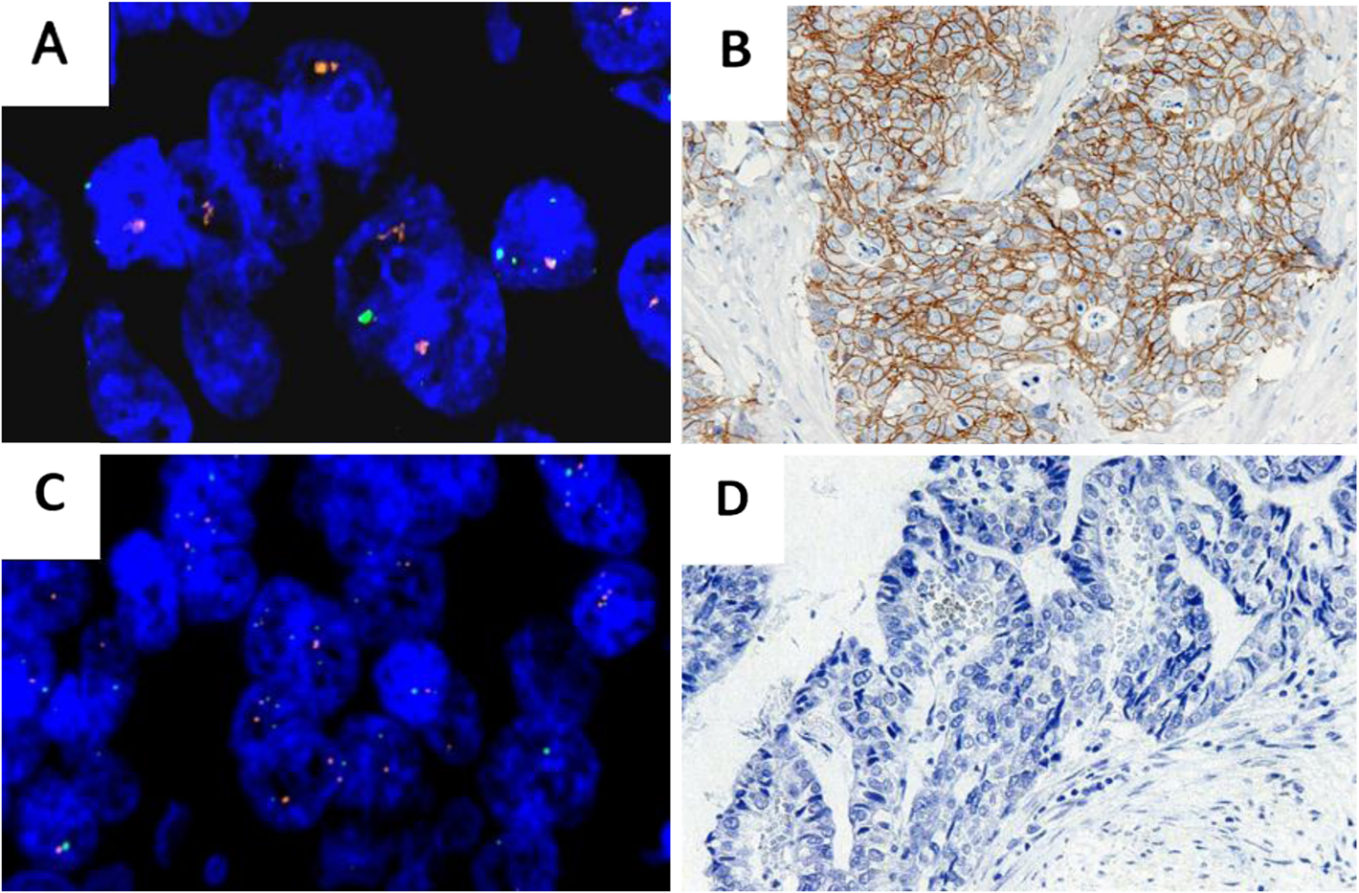
**HER2 expression as detected by IHC and FISH in discordant cases where HER2 status was positive in the LNM but negative in the PGC. (A)** FISH-positive LNM sample. **(B)** IHC-positive LNM sample. **(C)** FISH-negative PGC sample. **(D)** IHC-negative PGC sample.

**Figure 2:**
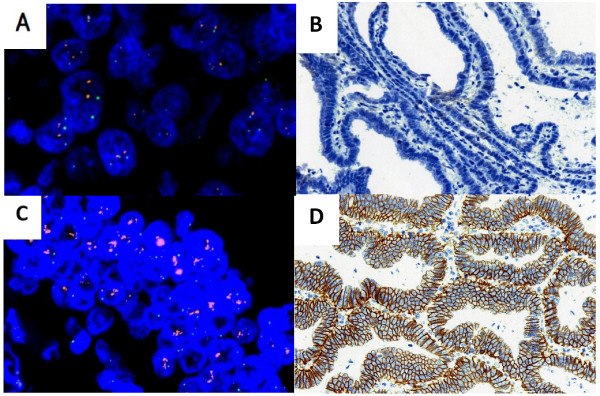
**HER2 expression as detected by IHC and FISH in discordant cases, where HER2 status was positive in the PGC but negative in the LNM. (A)** FISH-negative LNM sample. **(B)** IHC-negative LNM sample. **(C)** FISH-positive PGC sample. **(D)** IHC-positive PGC sample.

Of the 22 suitable PGC and LNM samples showing a result of 2+ by IHC or a discordance in ICH results between PGC and LNM, 12 of the PGC specimens (54.5%) showed positive HER2 expression, while 11 of the LNM samples (50.0%) showed positive (+2) HER2 expression as detected by FISH. Of the 102 PGC samples, 27 (26.5%) showed positive HER2 expression as detected by FISH + IHC, while 29 of 102 LNM samples (28.4%) showed positive HER2 expression. Overall, the general pattern of HER2 protein expression in PGC as assessed by FISH + IHC was the same as in its corresponding LNM (Table 3). The HER2 amplification status was evaluable by FISH + IHC in 102 paired PGC and LNM specimens: 23 (22.5%) were amplified and 69 (67.6%) were unamplified at both sites. There were 4 discordant cases (3.9%), where HER2 status was positive in the PGC but negative in the LNM (Figure 1), and 6 discordant cases (5.9%), where HER2 status was positive in the LNM but negative in the PGC (Figure 2). The results of FISH + IHC showed very strong concordance in HER2 status between the PGC and LNM groups (*k* = 0.754).

**Table 3:** Comparison of HER2 status as assessed by IHC and FISH in 102 primary gastric carcinomas and their corresponding lymph node metastases

	**LN metastatic site**					
	**Negative**	**%**	**Positive**	**%**	**Total**	**%**
**Primary tumor**						
Negative	69	67.6	6	5.9	75	73.5
Positive	4	3.9	23	22.5	27	26.5
Total	73	71.6	29	28.4	102	100

*k* = 0.754.

## Discussion

In the ToGA trial, the first phase III study of trastuzumab in conjunction with standard chemotherapy, HER2-positive patients with advanced gastric or gastro-esophageal junction cancer were randomized to receive 5-fluorouracil/capecitabine and cisplatin, either alone or in combination with trastuzumab. Based on the results of that study, HER2-targeted molecular therapies acquired high relevance in the treatment of gastric cancer [12], indicating the importance of identifying which clinical and/or pathological features or molecular differences might be predictive of sensitivity or resistance to anti-HER2 therapies.

Amplification of the HER2 gene and over-expression of its protein in gastric cancer were first described in 1986 [17], since which a number of studies have confirmed those findings [18-20]. However, how differing deregulation of HER2 between the primary gastric cancer and its metastases affects its potential as a prognostic factor remains to be clarified. Axillary lymph node metastasis is one of the most important prognostic determinants in breast carcinoma. The histopathologic characteristics and expression of biological markers vary among the same histologic subtypes of breast carcinoma. This suggests that the specific clinical and histopathologic features of the primary tumor and axillary lymph node metastases such as to a sentinel node might be used to tailor loco-regional and systemic treatment in different clinical settings in breast cancer [13-15].

Although molecular patterns may differ between a primary tumor and its associated metastases, patterns used to predict response to a given agent are usually derived from those within the primary tumor, with little attention to those found at other sites. Only a few studies have been published on this subject. Some studies assessed HER2 status in gastric cancer and confirmed a high level of concordance between HER2 status in the PGC and at various metastatic sites [20-23]. However, another study reported discordant data between HER2 status in the PGC and at various metastatic sites [24]. However, given the low number of cases analyzed and the types of cytological analysis used in these earlier studies, it is difficult to draw any conclusion with regard to HER2 status in synchronous and metachronous metastases.

The concept that distant metastases may indeed show molecular patterns which differ from those in the primary tumor is supported by our current assessment of HER2 gene status by IHC and FISH. The present results showed only a moderate correlation between HER2 gene status in the PGCs and that in the LNMs. Therefore, HER2 evaluation by IHC and FISH performed only on the primary tumor may not be accurate enough to select candidates for targeted therapy. The heterogeneity of HER2 gene status in neoplastic tissues obtained from different sites in the same patient is confirmed by our findings in synchronous LNMs, with a number of patients showing a difference in HER2 gene status between the primary tumor and its distant metastasis: in 4 cases (3.9%) the HER2 gene status was observed to be positive in the PGCs and negative in the LNMs, while in 6 cases (5.9%), HER2 gene status was positive in the LNMs and negative in the PGCs. It is important to note, however, that concordance between the PGCs and LMNs was found in 92 of the 102 patients included in this study (90.2%). This suggests that the treatment policy could be decided based on a sample of the PGC alone in cases where there are recurrent metastases.

Even though treatment with trastuzumab is effective, the decision to treat with trastuzumab is currently based on HER2 assessment in the PGC alone. However, the present results suggest that, as much as possible, the decision to treat with trastuzumab should be based on HER2 status as assessed in metastatic lesions.

The samples obtained in this study were obtained as far back as 1990, which means that some of them were unsuitable for application of FISH, although IHC was possible in all. To our knowledge, this is the first study including a large sample of HER2-positive patients to investigate the effect of molecular differences on the potential of HER2 status as a prognostic marker in PGCs with synchronous LNMs.

## Conclusion

The results of this study indicate that HER2 status in the PGC is a reliable basis for deciding whether to treat with anti-HER2 agents in patients with LNMs. Further prospective studies are needed, however, to determine the validity of these findings. We believe that these results may be of use in clinical practice and should be taken into account in designing future clinical trials based on anti-HER2 therapy.

## Consent

Written informed consent was obtained from the patients for publication of this report and any accompanying images.

## Competing interests

The authors declare that they have no competing interests.

## Authors’ contributions

MK, MS, and TT designed the study and wrote the manuscript. SM analyzed the histological slides. YM, TF, HT, MW, and HS collected the patients’ clinical information and obtained the follow-up data. All authors have read and approved the final manuscript.
